# Efficacious Recombinant Influenza Vaccines Produced by High Yield Bacterial Expression: A Solution to Global Pandemic and Seasonal Needs

**DOI:** 10.1371/journal.pone.0002257

**Published:** 2008-05-21

**Authors:** Langzhou Song, Valerian Nakaar, Uma Kavita, Albert Price, Jim Huleatt, Jie Tang, Andrea Jacobs, Ge Liu, Yan Huang, Priyanka Desai, Gail Maksymiuk, Virginia Takahashi, Scott Umlauf, Lucia Reiserova, Rodney Bell, Hong Li, Yi Zhang, William F. McDonald, T. J. Powell, Lynda Tussey

**Affiliations:** 1 VaxInnate Corporation, Cranbury, New Jersey, United States of America; 2 VaxInnate Corporation, New Haven, Connecticut, United States of America; University of California San Francisco, United States of America

## Abstract

It is known that physical linkage of TLR ligands and vaccine antigens significantly enhances the immunopotency of the linked antigens. We have used this approach to generate novel influenza vaccines that fuse the globular head domain of the protective hemagglutinin (HA) antigen with the potent TLR5 ligand, flagellin. These fusion proteins are efficiently expressed in standard *E. coli* fermentation systems and the HA moiety can be faithfully refolded to take on the native conformation of the globular head. In mouse models of influenza infection, the vaccines elicit robust antibody responses that mitigate disease and protect mice from lethal challenge. These immunologically potent vaccines can be efficiently manufactured to support pandemic response, pre-pandemic and seasonal vaccines.

## Introduction

Influenza is one of the major infectious disease threats to the human population. It affects individuals of all ages, causes repeated infections throughout life, and is responsible for recurrent seasonal epidemics as well as periodic global pandemics of varying severity. Vaccines are central both to the effective control of seasonal outbreaks and to pandemic preparedness. Hemagglutinin (HA) has been the key protective antigen in seasonal influenza vaccines for the last forty years. While its structure and the basis of its efficacy are well understood, the genetic variability of HA coupled with current methods of vaccine production make it exceedingly difficult to simultaneously meet seasonal and pandemic needs on a global basis. HA changes antigenically to evade the immune response and on average, the prevalent influenza strains in circulation will acquire three to four amino acid changes per year in HA, mostly in regions of HA that are recognized by protective antibodies. Mutations accumulate over time and approximately every three to five years the virus evolves into an antigenically distinct strain [1]. This requires regular updates of the vaccine strains. Additionally, influenza vaccines are typically produced in eggs via a process that takes place nearly year round. Therefore, worldwide production capacity for influenza vaccines is continuously dedicated to the production of seasonal vaccines while pandemic preparedness, either in response to an emerging pandemic or for the generation of a stockpile, requires the redirection of manufacturing resources to the production of a pandemic vaccine at the expense of the seasonal vaccine.

The current inter-related nature of seasonal and pandemic vaccine production has led to intense interest in the development of innovative technologies which could support both seasonal and pandemic influenza vaccine production. Improvements in influenza vaccine production by the industry have recently focused on cell culture. This approach alleviates the significant manufacturing issues associated with egg based manufacturing, but does not improve production efficiency. The intense focus on cell culture production stems from the historical view that protective forms of HA antigens must be manufactured using eukaryotic cells, like those of humans and chickens. The reason for this is that HA undergoes host cell dependent post-translational modification and even though the location and number of different glycosylation sites are not conserved among HAs, it is thought that glycosylation aids in correct folding of the molecule [2]. More recent data, however, show that the glycosylation pattern of HA does not impact the antibody response, suggesting that glycosylation is not required for appropriate folding of the molecule [3].

In addition to improvements in vaccine production efficiency, enhancement of the immunopotency of influenza vaccines will be required in order to meet seasonal and pandemic needs on a global scale. It is now well established that physical linkage of Toll-like receptor (TLR) ligands and vaccine antigens enhances the immunopotency of the linked antigen. TLRs are expressed on various cell types, including professional antigen presenting cells (APC), where they act as primary sensors of microbial infection and then activate signaling pathways that lead to the induction of immune and inflammatory genes. TLR agonists are molecules such as lipoproteins, lipids, sugars or nucleic acids that are specifically associated with pathogenic organisms. Engagement of TLRs by their cognate agonists and the subsequent signaling within APC leads to enhanced processing and presentation of antigens that are co-delivered to those APC [4], [5]. Recently, we demonstrated that the physical linkage of vaccine antigens to the Toll-like receptor 5 (TLR5) ligand, flagellin, generates a significantly more potent vaccine than simple mixing of antigen and flagellin[6], [7], [8].

We here present an approach that addresses many of the production and immunopotency barriers currently associated with seasonal and pandemic influenza vaccines. We have identified a single domain based on the globular head domain of HA which is a self-sufficient protective subunit that can be produced using prokaryotic expression systems. This globular head domain spans the majority of the neutralizing epitopes in HA and stably refolds to faithfully form these conformationally sensitive epitopes. We have genetically fused the globular head subunit to the TLR5 ligand flagellin to create an immunologically potent, highly protective vaccine that is very efficiently manufactured. The increased production efficiency associated with these vaccines means that they can be produced to meet national and even global needs in a period of several months with minimal investments in manufacturing infrastructure.

## Results

### Rational Design of Globular Head Constructs

**Figure 1:**
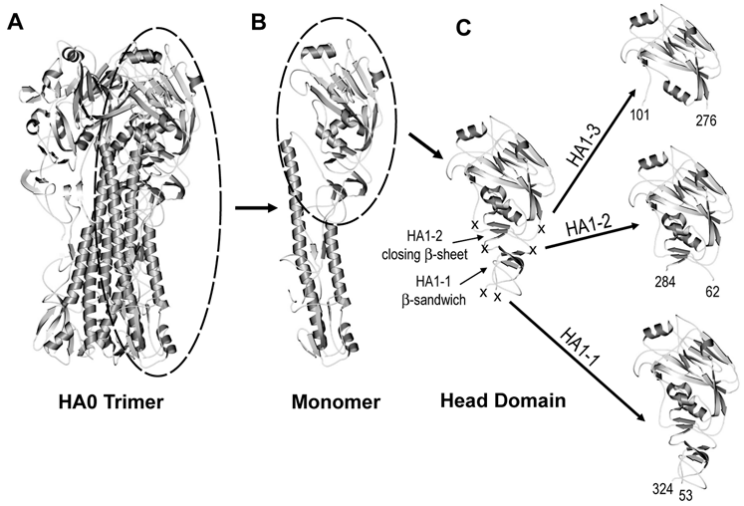
Depiction of the HA1-1, HA1-2 and HA1-3 globular head subunits. A) Ribbon diagram of the trimeric PR8 HA0 ectodomain with a monomeric subunit of the HA trimer circled. B) Ribbon diagram of a monomer with the globular head circled. C) Ribbon diagram of the globular head with the boundaries of the HA1-1, HA1-2 and HA1-3 constructs indicated by crosses. Each construct is presented in detail to the right. The beginning and ending residue numbers, in PR8, for the three constructs are labeled. The important secondary structure elements, such as β-sandwich in HA1-1 and the closing β-sheet in HA1-2 are also marked.

Structural studies have shown that two polypeptides, HA1 and HA2, form the monomeric subunit of the HA trimer. The HA1 polypeptide extends up from a membrane proximal stalk, spans the globular head domain and then returns to the stalk. Based on the architecture of HA1, we designed a subunit vaccine which encompassed the neutralizing epitopes of the globular head and also contained the structural elements necessary for spontaneous and efficient folding to correctly display these epitopes after recombinant protein expression in *E.coli*. The X-ray crystal structure of A/Puerto Rico/8/34 (PR8) was used to guide the design of three prototypic constructs [9] (Figure 1 A–C). In the PR8 crystal structure the HA1 polypeptide has a long tail that interacts with the central coiled-coil formed by the HA2 subunit. The tail is loose and extended, a structural feature that could complicate expression and refolding if included in a subunit construct. By comparison, the globular head domain of HA1 is compactly folded. The head is formed by multiple secondary structural elements that include a β-pleated sheet on the top of the head with tightly packed short α-helices underneath. A second, three-stranded β-sheet lies underneath the α-helices on the membrane-proximal side. An additional small β-sandwich is situated underneath this second β-sheet. Non-structured peptides connect the second β-sheet and the small β-sandwich. These linking peptides do not function as stabilizing elements to the tertiary structure of the globular head, and thus provide ideal sites to truncate the globular head from the rest of HA1 such that it folds independently. We hypothesized that interactions between the α-helices and the second β-sheet, aided by a disulfide bond between the third and fourth conserved cysteines of HA1, are the minimal structural elements necessary for the stable, independent folding of the head domain and maintenance of native, neutralizing epitopes. Therefore, in the design of the first prototypic construct, HA1-2, the boundary was placed in these linking peptides at residues K62 on the N-terminus and S284 on the C-terminus (Figure 1C). Two conserved disulfide bonds are preserved in this molecule.

We also recognized that inclusion of the small β-sandwich could be required to further stabilize the globular head domain subunit. Therefore, the second prototypic construct, designated as HA1-1, includes this additional secondary structure, and is similar to the thermolysin released fragment previously described by Bizebard *et al* [10]. The domain boundary for HA1-1 (PR8) was placed between residues S53 and R324 (Figure 1C). The resulting construct contains four conserved disulfide bonds.

In order to evaluate the importance of secondary structural elements such as the second β-sheet and the small β-sandwich in the stabilization of the independent head domain and the consequent display of conformational epitopes, we designed a third PR8 construct, HA1-3, as a control. The boundary for HA1-3 (PR8) was placed at residues N101 to G276 to form a construct which is similar in design and size to an HA subunit previously reported by Jeon and Arnon [11]. The HA1-3 boundary eliminates the β-sheet underneath HA1-2 and all but one conserved disulfide bond.

Each of the globular head constructs were recombinantly linked to the C terminus of *Salmonella typhimurium* type 2 flagellin (STF2) and the resulting fusion proteins were designated as STF2.HA1-1 (PR8), STF2.HA1-2 (PR8) and STF2.HA1-3 (PR8) according to their decreasing length.

### Expression and Conformational Integrity of Flagellin Fusion Proteins

**Figure 2:**
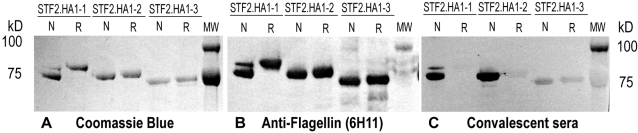
STF2.HA1-1, STF2.HA1-2 and STF2.HA1-3 expression, purification and immmunoreactivity. STF2.HA1-1, STF2.HA1-2 and STF2.HA1-3 proteins were expressed and purified. Refolded proteins were analyzed by SDS-PAGE and Western blot analyses. A) Coomassie stained gel showing the proteins run in the presence (R) or absence (N) of reductant. Bands of the appropriate molecular weight were observed for each construct. B) Western blot analyses using the anti-flagellin monoclonal antibody, 6H11. C) Western blot using PR8-specific immune sera raised following a sub-clinical infection of mice with the PR8 virus.

The STF2.HA1-1 (PR8), STF2.HA1-2 (PR8) and STF2.HA1-3 (PR8) fusion proteins were expressed using standard *E. coli* cell culture. All three proteins expressed equally well following induction. Purified protein was denatured, refolded by rapid dilution and analyzed by SDS-PAGE and western blot under reducing and non-reducing conditions. The Coomassie stained gels show that the purified STF2.HA1-2 (PR8) and STF2.HA1-3 (PR8) proteins were homogeneous. STF2.HA1-1 (PR8) had a predominant band migrating at the correct size and a minor band migrating at a higher apparent molecular weight (Figure 2A).

To evaluate the conformational integrity of the HA subunit of the fusion proteins, Western blots of the SDS-PAGE gels were probed with either the flagellin-specific monoclonal antibody, 6H11, which recognizes a linear epitope (Figure 2B) or convalescent mouse sera raised following a low dose challenge of mice with PR8 virus (Figure 2C). Reactivity of the convalescent sera with protein run in the absence versus the presence of reductant was used as a measure of conformational integrity of the HA moiety. The results show that while 6H11 reacted equally well with all proteins before and after the addition of reductant, the PR8 convalescent serum reacted strongly with the non-reduced forms of STF2.HA1-2 (PR8) but very weakly with the reduced forms, indicating that the majority of epitopes recognized by HA-specific antibodies in the convalescent anti-sera are conformational, and reliant on disulfide bonding. The residual reactivity with the convalescent sera following the addition of reductant is likely due to the small portion of polyclonal antibodies that recognize linear epitopes rather than conformational epitopes. These results demonstrate that the globular head domain correctly refolds during the refolding and purification of STF2.HA1-2 (PR8) protein. A similar pattern of reactivity was observed for the major band of STF2.HA1-1 (PR8). Interestingly, the minor, slower-migrating STF2.HA1-1 band co-migrates with the reduced form of the protein and reacts poorly with the convalescent sera, suggesting that this band corresponds to misfolded protein. In contrast to STF2.HA1-1 and STF2.HA1-2, the convalescent sera reacted poorly with both the non-reduced and reduced forms of STF2.HA1-3 (PR8), indicating that the globular head domain in this recombinant protein is misfolded under the conditions tested. This is consistent with our hypothesis that secondary structures positioned near the peptides linking the globular head domain to the stalk are likely required to ensure stable refolding of the molecule.

**Figure 3:**
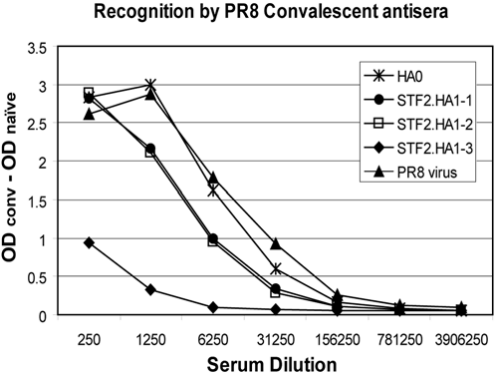
Immunoreactivity of STF2.HA1-1, STF2.HA1-2 and STF2.HA1-3 in ELISA. ELISA plates were coated with the 0.2 µg/well of indicated STF2 fusion proteins, PR8 virus or the full length PR8 HA0 ectodomain expressed in Hi5 cells. Plates were probed with either naïve or PR8 convalescent sera at indicated dilution. Following incubation with HRP-conjugated goat anti-mouse IgG, plates were developed with UltraTMB substrate. Results reflect the delta value of OD_450_ (Convalescence-Naïve) of samples performed in duplicate. Naïve values (data not shown) were below 0.02.

To further evaluate the conformational integrity of the HA moiety of the STF2.HA1-1 (PR8), STF2.HA1-2 (PR8) and STF2.HA1-3 (PR8) fusion proteins, ELISA plates were coated with serial dilutions of the full length HA (ecto-domain, HA0, produced in Hi5 cells), PR8 virus and the fusion proteins. The coated plates were probed with either naïve or convalescent mouse sera (Figure 3). As expected, none of the proteins reacted with naïve sera (data not shown). PR8 convalescent sera reacted with PR8 virus and ecto-domain HA0 slightly better than STF2.HA1-1 and STF2.HA1-2 due to their additional epitopes. STF2.HA1-1 was equally antigenic as STF2.HA1-2 despite the minor contamination of the slower migrating band. Consistent with the western blot results shown in Figure 2C, STF2.HA1-3 demonstrated the least reactivity with the convalescent sera.

An *in vitro* assay based on a cell line that expresses TLR5 and secretes TNF-α in response to TLR signaling was used to assess the functional integrity of the flagellin moiety. In this assay, all three fusion proteins induced strong TNFα secretion, indicative of potent TLR5 activity (EC_50_≤10 ng/ml for each protein). The endotoxin level for each protein was less than 0.05 EU/µg as measured by LAL assay. These results demonstrate that the flagellin moiety remains functional in the context of the fusion protein.

Taken together, the results confirm the importance of secondary structure in the appropriate refolding of the HA globular head domain. While both the STF2.HA1-1 and STF2.HA1-2 protein fold properly, the STF2.HA1-2 recombinant protein folds more efficiently under the conditions tested. The globular head component of STF2.HA1-3 fails to fold efficiently.

### Conformational Integrity of Neutralizing Epitopes

**Figure 4:**
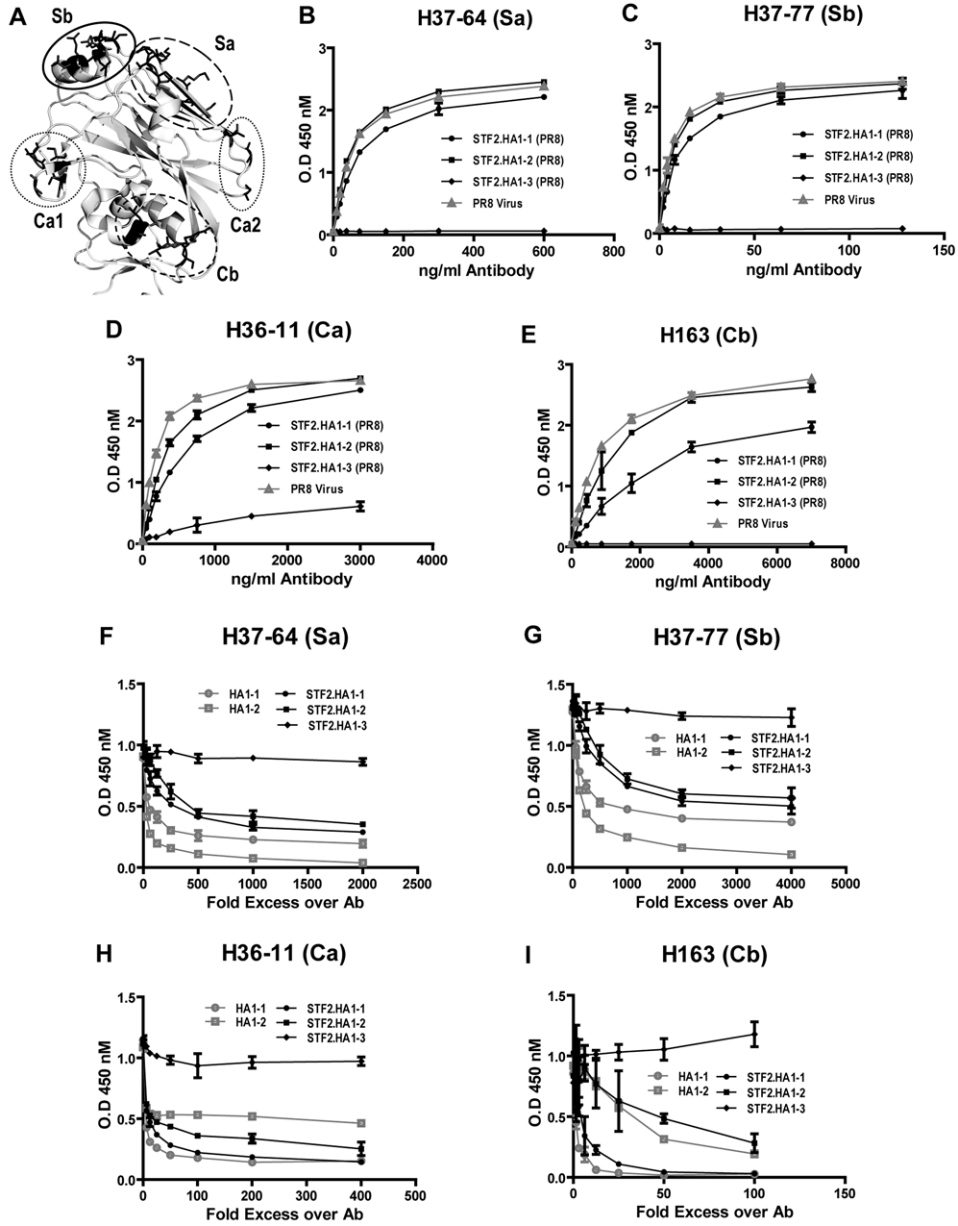
Comparative recognition of STF2.HA1-1, STF2.HA1-2, STF2.HA1-3 and PR8 virus by neutralizing monoclonal antibodies. A) Ribbon diagram depicting of the known antigenic regions of the HA globular head. B–E) ELISA plates were coated with either PR8 virus or the STF2 fusion proteins and probed with a mAb specific for the Sb, Sa, Ca1, Ca2 and Cb region. All STF2 fusion proteins are produced from E coli. F–I) ELISA plates were coated with STF2.HA1-2 and competed against soluble form of HA1-1, HA1-2, STF2.HA1-2, STF2.HA1-3 in binding of the panel of monoclonal antibodies (H37-64, 18 ng/ml; H37-77, 8 ng/ml; H36-11, 188 ng/ml and H163, 500 ng/ml). Bound antibodies were detected by 450 nm absorption. HA1-1 and STF2.HA1-1 were produced in insect cell culture. Both proteins have C-terminal 6His tag. HA1-2, STF2.HA1-2 and STF2.HA1-3 were produced in E. coli. HA1-2 has 6His tag at C-terminus.

The conformational integrity of defined antigenic regions of the PR8 globular head was tested using a panel of monoclonal antibodies known to be specific for neutralizing epitopes located in the globular head [12], [13]. ELISA plates were coated with either PR8 virus or the fusion proteins STF2.HA1-1 (PR8), STF2.HA1-2 (PR8) or STF2.HA1-3 (PR8). Plates were probed with neutralizing monoclonal antibodies representing each of four previously defined antigenic regions of PR8 (Sa, Sb, Ca, Cb, Figure 4A). As shown in Figure 4B-4E the STF2.HA1-1 (PR8), STF2.HA1-2 (PR8) proteins and the influenza virus reacted comparably with the panel of monoclonal antibodies. There is some reduction in reactivity for the STF2.HA1-1 (PR8) protein which is likely due to the presence of misfolded molecules in the preparation, in agreement with the western blot and convalescent serum ELISA data presented above. The STF2.HA1-3 (PR8) protein failed to bind the monoclonal antibodies.

The structural integrity of these neutralizing epitopes was further examined using competition assays. Since the panel of monoclonal antibodies reacted equally well with plate-bound STF2.HA1-2 (PR8) and virus particles, the ELISA plates were coated with the STF2.HA1-2 (PR8) fusion protein. The panel of monoclonal antibodies was incubated with the HA1-2 globular head protein alone, STF2.HA1-2 or STF2.HA1-3 for 2 hours. HA1-1 globular head alone or the STF2.HA1-1 protein produced in baculovirus (HA1-1 bv) were included in the evaluation. The mixture was transferred to the ELISA plates and the specific monoclonal antibody reactivity was measured following washing and blocking of the plates. The results (Figure 4F–I) show that HA1-1bv, STF2.HA1-1bv, HA1-2, and STF2.HA1-2, but not STF2.HA1-3 proteins compete for binding of the monoclonal antibodies to plate-bound STF2.HA1-2. These results demonstrate that HA1-1 and HA1-2, either in the context of the fusion or expressed alone, fold properly and that the neutralizing epitopes are correctly displayed. The results further confirm that the STF2.HA1-3 protein fails to stably refold.

### Immunogenicity and Efficacy of STF2.HA1-2 in Mice

**Figure 5:**
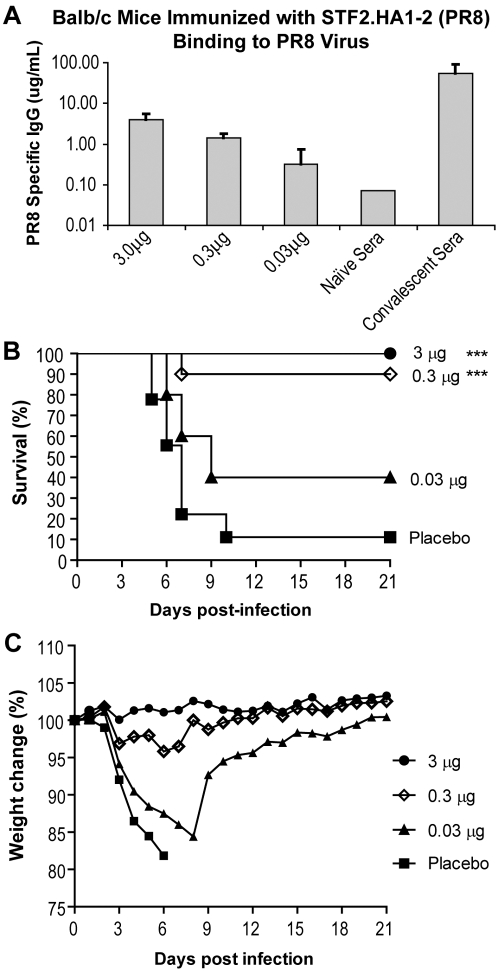
STF2.HA1-2 mediated protective immunity against lethal challenge *in vivo*. BALB/c mice (10/group) were immunized on day 0 and 14 with 3, 0.3 or 0.03 µg of STF2.HA1-2. A group receiving formulation buffer alone was included as a negative control. A) Sera were harvested on day 21 and evaluated for HA-specific antibody responses by ELISA. B–C) On day 28, mice were challenged *i.n.* with 1×LD_90_ of influenza PR8. Survival (B) and weight loss (C) of individual mice were monitored for 21 days post-challenge.

Groups of 10 BALB/c mice were immunized on days 0 and 14 with 3, 0.3 and 0.03 µg of STF2.HA1-2. A group of naïve mice was included as a negative control. On day 10, animals were bled and the sera of individual animals examined for HA-specific IgG by probing ELISA plates coated with the PR8 virus (Figure 5A). Pooled convalescent sera were included as a positive control. The results demonstrate that immunization with as little as 0.03 µg of STF2.HA1-2 induced measurable levels of HA-specific antibodies post the priming immunization. Mice were subsequently challenged on day 28 with 1×LD_90_ of the mouse-adapted influenza PR8 virus delivered intranasally. Survival and weight loss were followed for 21 days (Figure 5B and C). As shown in Figure 5B, naïve mice exhibited signs of infection as early as 4 days post-challenge and 90% of the animals succumbed to the lethal challenge by day 21. In contrast, 100% of mice immunized with 3 µg of STF2.HA1-2 (PR8) and 90% of mice immunized with 0.3 µg of STF2.HA1-2 (PR8) survived the challenge. Mice immunized with 3 µg of STF2.HA1-2 (PR8) exhibited no signs of disease as measured by weight loss while mice immunized with as little as 0.3 µg exhibited only very mild weight loss (Figure 5C). Forty percent of mice immunized with 0.03 µg were protected against the lethal challenge. These results demonstrate that *E. coli* expressed STF2.HA1-2 induces an HA-specific immune response that successfully protects BALB/c mice from a lethal challenge with influenza A virus.

### Application to a Currently Circulating Influenza A Strain

The same principles of design were applied to the HA of the currently circulating seasonal strain A/Solomon Islands/3/2006 (SI). Protein was expressed and purified using the methodologies as for the prototypic PR8 construct. To evaluate the immunogenicity and efficacy of this construct, groups of 10 BALB/c mice were immunized on day 0 and day 14 with 3 or 0.3 µg of STF2.HA1-2 (SI), and bled on day 21. Sera were analyzed for hemagglutination inhibition of SI virus using chicken red blood cells as the target. Geometric mean titers (GMT) were 1∶320 (range 1∶160–1∶640) at the 3 µg dose level and 1∶226 (range 1∶80–1∶640) at the 0.3 µg dose level. Ferret immune sera, raised on natural infection and obtained from CDC, exhibited an HAI titer of 1∶320. To further characterize the potency of the vaccine, 15 BALB/c mice were immunized twice with 10, 1 or 0.1 µg of STF2.HA1-2 (SI). Sera were harvested 1 week post the boosting immunization and evaluated for microneutralization titers. In instances where a mouse adapted strain of the virus is not available, as is the case for this seasonal strain, neutralization titers of ≥1∶40 are generally accepted as a correlate of efficacy. The results are reported as GMT in Table 1. The results show that doses of 1 µg elicit GMT titers well above 1∶40 in the mouse model.

**Table 1:** Neutralization of Influenza A/Solomon Islands/3/06 by Mouse and Rabbit Immune Sera

Groups [N]	Dose a	GMT b	95% CI c
**Mouse**			
STF2.HA1-2 (SI) [10]	10	845***	472–1511
STF2.HA1-2 (SI) [14]	1	297***	106–833
STF2.HA1-2 (SI) [10]	0.1	8	4–20
Naive		5	5–5
**Rabbit**			
STF2.HA1-2 (SI) [6]	15	640***	404–1014
STF2.HA1-2 (SI) [6]	5	453***	211–971
Naïve [6]		40	40–40

a µg/animal;

b geometric mean titers;

c 95% confidence intervals;

*** , *p*<0.001, significance **vs** naïve in ANOVA/Tukey Multiple Comparison Test.

To further evaluate the immunopotency of STF2.HA1-2 (SI), groups of 6 New Zealand White rabbits were immunized twice, *i.m*., with 15 or 5 µg of STF2.HA1-2 (SI). Sera were harvested 3 weeks post the boosting immunization and tested for microneutralization titers. The results in Table 1 demonstrate that STF2.HA1-2 (SI) elicits robust virus neutralizing titers in the rabbit model. Ferret immune sera, raised on natural infection and obtained from the CDC, was included as a positive control in these assays and found to have a neutralizing titer of 1∶5,120.

## Discussion

We have identified the structural elements necessary for efficient refolding of a protective HA subunit after recombinant protein expression in *E.coli*. Structural information is available for the H1 [9], H3 [14], H7 [15], H5 and H9 subtypes [16] of influenza A as well as for an influenza C HEF (hemagglutinin, esterase, and fusion glycoprotein) [17]. Comparisons of these structures show that the different HAs, while antigenically distinct, are structurally similar and share a common sub-domain organization. Each HA monomer folds to form a membrane proximal stalk and a membrane distal globular head domain. The globular head stands independently from the central stalk and contains the majority of the neutralizing antibody epitopes [12], [13]. We designed three PR8 HA prototypic constructs to test the hypothesis that secondary structures such as the β-pleated sheets near these linker peptides are sufficient to support the head domain as a self-stabilizing unit that can be engineered and expressed apart from the rest of the molecule.

We find that of the three prototypic constructs evaluated, STF2.HA1-2 provides the level of expression and ease of conformationally correct refolding required to support a truly efficient, scalable manufacturing process. This molecule expressed well in our prokaryotic system and refolded easily using rapid dilution. STF2.HA1-1 also refolded albeit with somewhat lower efficiency than STF2.HA1-2, presumably as a consequence of the additional domain and two additional disulfide bonds. Western blots of STF2.HA1-1 run under reducing and non-reducing conditions and probed with PR8 convalescent sera reveal a band in the non-reduced sample that co-migrates with the main band in the reduced sample suggesting that a significant proportion of the protein remained misfolded. STF2.HA1-3 refolded the least efficiently, most likely due to the absence of secondary structures required for stable refolding. Reactivity with a panel of defined neutralizing monoclonal antibodies further supports this conclusion.

STF2.HA1-2 (PR8) was found to be highly immunogenic and efficacious against a lethal challenge in the mouse model. Mice receiving doses of STF2.HA1-2 (PR8) as low as 0.3 µg were protected against a lethal challenge of virus. These data demonstrate that a subunit of HA based on the globular head domain can be fully protective in a standard mouse lethal challenge model. When the same principles of design were applied to the currently circulating seasonal strain, A/Solomon Islands/3/2006, we found that STF2.HA1-2 (SI) was highly immunogenic in both mice and rabbits. In mice doses of 1 µg elicited geometric mean neutralizing titers of 1∶297 and in rabbits doses of 5 µg elicited titers of 1∶453. Thus, the principles of design for these protective subunit vaccines can be applied to different HA molecules.

A key benefit with this approach is that the STF2.HA1-2 recombinant fusion protein can be made quickly, inexpensively and in quantities sufficient to meet global needs. The efficiency of this technology translates approximately into a 1,000 fold gain in production. As a point of reference, the average yield for cell culture is 3 mg/L; for egg based production, 7 mg/L; for baculovirus recombinant synthetic protein, 13 mg/L and for the standard prokaryotic system described here, 3,700 mg/L. This increase in production capacity, along with the fact that it is carried out in a prokaryotic system, provides an opportunity to address several shortcomings of the current egg-based system. One advantage deriving from increased capacity is the ability to increase the dose of antigen. Studies have shown that persons greater than 65 years of age respond less well to the standard vaccine, and that increasing the dose of HA four to five-fold substantially improves the immune response in this segment of the population. Formulation of a “high-dose” vaccine for the elderly becomes a practical possibility with an unconstrained supply of antigen. A second set of advantages comes from eliminating the growth of virus from the manufacturing process. Currently, vaccine production strains are created by crossing the HA and NA genes from candidate circulating strains onto an egg-adapted virus, generally the PR/8/34 strain. Manufacturers then further adapt these production strains to create high-yield viruses. The adaptation process results in selection of mutations in the upper part of the HA globular head near the receptor binding site [18]. As a consequence, the HA in the vaccine may be different from the HA in the original viral isolate. Prokaryotic expression, based on a cDNA copy of the original isolate HA, avoids genetic selection during the production process. In addition, there are situations where a suitable production strain cannot be created by conventional methods. In these cases, a related HA production strain is pressed into service. Eliminating the need to grow virus allows the production of the desired antigen with the original sequence.

In conclusion, the manufacturing approach described herein has major advantages over existing technologies in that it allows faster molecular development, rapid manufacturing and very high levels of productivity at small manufacturing scales. These advantages are critical to the successful production of seasonal and pandemic influenza vaccines.

## Materials and Methods

### Cloning of recombinant HA genes

#### E. coli

The codon optimized synthetic genes of the hemagglutinin (HA) globular head domain of PR8 were fused to the C-terminus of the full-length sequence of *Salmonella typhimurium* fljB (flagellin phase 2), STF2 (DNA2.0 Inc., Menlo Park, CA). The sequence SGSGSGS was incorporated at the junction of STF2 and HA as a flexible linker. The resulting fragments corresponding to the STF2.HA1-1, 1-2 and 1-3 genes respectively were cloned to pET24a vector to generate the constructs STF2.HA1-1, STF2.HA1-2 and STF2.HA1-3. The proteins were expressed in BLR3 (DE3) cells (Novagen, San Diego, CA; Cat #69053).

#### Baculovirus

The synthetic genes encoding HA1-1 (PR8) or in fusion with STF2 were codon-optimized for Baculovirus expression (DNA2.0 Inc., Menlo Park, CA) and cloned into pFastBac™. The honey bee mellitin sequence (MKFLVNVALVFMVVYISYIYAD PS) was fused to the amino terminus of recombinant proteins to provide a secretion signal and hexahistidine was tagged to the carboxyl terminus to facilitate purification. The synthetic genes were cloned to the pFastbac1 vector. The recombinant Baculovirus generation followed standard Bac-to-Bac® Baculovirus Expression protocol (Invitrogen, Carlsbard, CA).

### Protein production

High expresser clones were cultured overnight and used to inoculate fresh LB medium supplemented with 25 µg/ml kanamycin, 12.5 µg/ml tetracycline and 0.5% glucose. At an OD_600_ = 0.6 protein expression was induced with 1 mM IPTG for 3 h at 37°C. Cells were harvested by centrifugation (8,000g for 7 minutes) and disrupted by microfluidizer (18,000 psi). The inclusion body was washed with 1% Triton X100 and dissolved in 8 M urea. The filtered protein solution in 25 mM NaCl and 50 mM Acetate, pH 4.0 was applied to a SP Sepharose Fast Flow column (GE/Amersham). The fraction peak was eluted by salt gradient and buffer exchanged to 50 mM Tris, 25 mM NaCl and 8 M urea, pH 8.0. Protein refolding was achieved by rapid dilution (1∶10) into 100 mM Tris-HCl buffer (pH 8.0), and further purified by anion exchange (Source Q, GE/Amersham). For final polishing and endotoxin removal, a Superdex 200 gel filtration column (10/300 GL, GE/Amersham) was used. The protein peak was eluted using 100 mM Tris, 150 mM NaCl, 1% glycerol and 1% Na-deoxycholate elution buffer. Peak fractions were pooled, dialyzed against 1×PBS and stored at −80°C. For all 6xHis tagged proteins, the metal chelating column was employed. Protein was loaded to a Ni-NTA column equilibrated in 20 mM Tris, pH 8, 0.5 M NaCl and eluted in a gradient of 0–0.5 M imidazole. The target protein was further purified by size exclusion column (10/300 GL, GE/Amersham). The peak fractions were pooled, concentrated and dialyzed against 1×PBS. Aliqoted protein solution was stored at −80°C. Endotoxin contamination was assayed by using standard Chromogenic Limulus Amebocyte Lysate assay (Cambrex, Walkersville, MD) as directed by the manufacturer.

### ELISAs

Many aspects of the ELISA methods were held in common. ELISA plates were coated with the indicated proteins in PBS overnight at 4°C or one hour at room temperature. All washes between reagent addition steps were performed 3 times with 1X PBS/0.05% Tween-20. Plates were blocked with 200–300 µl/well of Assay Diluent Buffer (ADB; BD Pharmingen) for 1–3 hour at 23–27°C. After incubation with the indicated detection antibodies, HRP-labeled goat anti-mouse antibody (Jackson Immunochemical) diluted in ADB was added and the plates were incubated at 23–27°C for 1 hour. After adding TMB Ultra substrate (Pierce) and monitoring color development, the reaction was stopped with 1 M H_2_SO_4_ and OD_450_ was measured on a microplate spectrophotometer.

#### Protein ELISAs

Plates were coated with serial dilutions of proteins. After block, plates were probed with monoclonal antibody specific for flagellin (6H11; Inotek) or convalescent sera against PR8 virus overnight at 4°C.

#### Serum antibody determination

Plates were coated with 100 µl/well HA1-1 produced in insect cells in PBS (5 µg/ml). Dilutions of the sera in ADB were added (100 µl/well) and the plates were incubated overnight at 4°C.

#### Viral ELISA

Sucrose density gradient purified PR8 virus (Advanced Biotechnologies Inc.,) or STF2 tagged recombinant HA proteins were diluted to 4 µg/ml in 1X PBS and 100 µl coated in triplicates. After block, plates were incubated with 100 µl of HA-specific antibodies diluted in ADB at 25°C for 2.5 hours.

#### Competition ELISA

Plates were coated overnight with 100 µl/well STF2.HA1-2 (PR8) at 2 µg/ml. Antibodies were pre-incubated with serially diluted recombinant proteins for 2 hours at 25°C and added to the washed and blocked STF2.HA1-2 (PR8) coated plates for a further 2 hour incubation followed by detection antibody. The amount of antibody used for each epitope was pre-determined to be in the linear range of a saturation curve in ELISA.

### Cells and viruses

MDCK cells were obtained from ATCC and maintained in DMEM supplemented with 5% FBS, 2 mM L-glutamine, 100 units/ml Penicillin and 100 µg/ml Streptomycin. Influenza viruses, mouse adapted A/PR/8/34 and A/Solomon Islands/3/2006, were obtained from Dr. Y. Kawaoka (University of Wisconsin) and CDC, respectively, and propagated in either MDCK cells or 11-day old SPF embryonated hen's eggs (Charles River Laboratories, North Franklin, CT).

### TLR5 bioassay

The bioactivity of purified recombinant proteins was tested as previously described [6].

### Animal studies

BALB/c mice 6–8 weeks old were purchased from the Jackson Laboratory (Bar Harbor, ME) and housed in either the Yale University vivarium (New Haven, CT) or the Princeton University vivarium (Princeton, NJ). All studies were performed in accordance with the University Institutional Animal Care and Use Committees (IACUC). Recombinant proteins were prepared in one of two vehicles: PBS (phosphate-buffered saline) or formula F147 (10 mM L-histidine, 150 mM NaCl, 5% trehalose, 0.02% polysorbate 80, 0.1 mM EDTA, 0.5% ethanol, 10 mM Tris, pH 7.2). Vehicles were used interchangeably without detectable impact on the results. Mice were immunized subcutaneously (*s.c*.) on days 0 and 14. On days 13 (primary) and 21 (boost), individual mice were bled by retro-orbital puncture. Sera were harvested by clotting and centrifugation of the heparin-free blood samples.

Studies with female and male New Zealand White rabbits were performed at Covance Research Products (Denver, PA). Rabbits (6/group) were immunized intramuscularly (*i.m.)* on days 0 and 21 with 5 or 15 µg of STF2.HA1-2. Sera were harvested 3 weeks post the booster and evaluated for HA-specific microneutalization titers.

### Microneutralization assays

Serum samples were treated with receptor destroying enzyme II (RDE, Denka Seiken Co., Ltd., Tokyo, Japan) and co-cultivated with 100 TCID_50_ of influenza virus A/PR/8/34 or A/Solomon Islands/3/2006 for 1.5 hr in series dilution (duplicate). MDCK cells (4×10^4^ /well) in DMEM supplemented with 1% BSA, 20 mM HEPES, and 100 IU/ml Penicillin and 100 µg/ml Streptomycin were then added and incubated for 20 hours at 37°C. Cells were washed, fixed, air-dried and incubated with a monoclonal anti-influenza A nucleoprotein antibody (1∶2,000, clones A1 and A3, ATCC/BEI resources). Signals were detected by OD_450_. Virus back titration, positive serum control, virus controls (VC), and cell controls (CC) were included in the assay. The end point of virus neutralizing antibody for each serum was determined using 50% of specific signal = [(Average OD of VC wells)–(Average OD of CC wells)]/2+Average OD of CC wells. Values below this value are considered positive for neutralizing activity.

### Statistical analyses

The titers of neutralizing antibodies were transformed into natural logarithm, and subjected to ANOVA/Tukey tests. Survival curves between different groups were compared with Log-rank test. Data analysis used GraphPad Prism version 5.00 for Windows (GraphPad Software, San Diego California USA, www.graphpad.com).

### Influenza virus challenge of mice

To assess efficacy, mice immunized on days 0 and 14 as described above were challenged on day 35 by intranasal administration of 1×LD_90_ (dose lethal to 90% of mice; 1×10^3^ TCID50) of influenza A isolate, PR8. Animals were monitored daily for 21 days following the challenge for survival and weight loss.
